# MicroRNA-503 inhibits the G1/S transition by downregulating cyclin D3 and E2F3 in hepatocellular carcinoma

**DOI:** 10.1186/1479-5876-11-195

**Published:** 2013-08-22

**Authors:** Fenqiang Xiao, Wu zhang, Liming Chen, Fei Chen, Haiyang Xie, Chunyang Xing, Xiaobo Yu, Songming Ding, Kangjie Chen, Haijun Guo, Jun Cheng, Shusen Zheng, Lin Zhou

**Affiliations:** 1 Key Laboratory of Combined Multi-organ Transplantation, Ministry of Public Health; Key Laboratory of Organ Transplantation, Zhejiang Province, Hangzhou, Zhejiang, P.R. China; 2 Division of Hepatobiliary and Pancreatic Surgery, First Affiliated Hospital, School of Medicine, Zhejiang University, Hangzhou, P.R. China; 3 Institution of Cardiology, The First Affiliated Hospital, School of Medicine, Zhejiang University, Hangzhou, P.R. China

**Keywords:** Hepatocellular carcinoma, microRNA-503, Cyclin D3, E2F3, Overall survival, G1/S transition

## Abstract

**Background:**

Increasing evidence indicates that deregulation of microRNAs (miRNAs) is involved in tumorigenesis. Downregulation of microRNA-503 has been observed in various types of diseases, including cancer. However, the biological function of miR-503 in hepatocellular carcinoma (HCC) is still largely unknown. In this study we aimed to elucidate the prognostic implications of miR-503 in HCC and its pathophysiologic role.

**Methods:**

Quantitative reverse transcriptase polymerase chain reaction was used to evaluate miR-503 expression in HCC tissues and cell lines. Western blotting was performed to evaluate the expression of the miR-503 target genes. *In vivo and in vitro* assays were performed to evaluate the function of miR-503 in HCC. Luciferase reporter assay was employed to validate the miR-503 target genes.

**Results:**

miR-503 was frequently downregulated in HCC cell lines and tissues. Low expression levels of miR-503 were associated with enhanced malignant potential such as portal vein tumor thrombi, histologic grade, TNM stage, AFP level and poor prognosis. Multivariate analysis indicated that miR-503 downregulation was significantly associated with worse overall survival of HCC patients. Functional studies showed miR-503 suppressed the proliferation of HCC cells by induction of G1 phase arrest through Rb-E2F signaling pathways, and thus may function as a tumor suppressor. Further investigation characterized two cell cycle-related molecules, cyclin D3 and E2F3, as the direct miR-503 targets.

**Conclusion:**

Our data highlight an important role for miR-503 in cell cycle regulation and in the molecular etiology of HCC, and implicate the potential application of miR-503 in prognosis prediction and miRNA-based HCC therapy.

## Background

Hepatocellular carcinoma (HCC) is one of the most common human malignancies and the leading cause of cancer-related death worldwide [1-3]. The development and progression of HCC is a multistage process involving the deregulation of genes that are crucial to cellular processes, such as cell cycle control, cell growth, apoptosis and cell migration. Aberration of protein-coding genes has been demonstrated to play a critical role in the development of human cancers. Recently, an increasing number of reports have suggested that deregulation of non-coding genes, particularly microRNAs (miRNAs), is also closely associated with tumorigenesis [4,5].

miRNAs are a class of short, endogenous, non-coding RNAs known to negatively regulate the expression of protein-coding genes through binding to the 3′ untranslated regions (3′ UTRs) of target genes [6,7]. One-third of protein-coding genes in humans are reportedly regulated by miRNAs [8]. miRNAs are involved in the regulation of various biological processes, such as development [9], cell proliferation, apoptosis [10], and differentiation [11]. Additionally, deregulation of miRNAs has been observed in various types of human cancer [4,12-16]. Growing evidence indicates that miRNAs may function as oncogenes or tumor suppressor genes during tumor development and progression [5].

Study on miR-503 itself, a member of the miR-16 family, was seldom reported, especially in HCC, although there were some reports about the findings for the same miRNA family members. Liu *et al*. reported that the miR-16 family induces cell cycle arrest by downregulating the expression of CCND3, CCNE1 and CDK6 [17]. miR-195, another member of the miR-16 family ,suppressed G1/S transition of human HCC cells by targeting cyclin D1, CDK6, and E2F3 [18]. Lu et al. reported that miR-503 was significantly down-regulated in oral cancer cell lines compared with normal oral keratinocytes [19]. Zhou et al. reported that miR-503 induced a G1 arrest and inhibited proliferation in MHCCLM3 cells [20]. However, the specific signaling pathways regulated by miR-503 and the detailed mechanisms in tumorigenesis are still unknown.

Our study determined that downregulation of miR-503 occurred frequently in HCC tissues and cell lines. miR-503 downregulation was correlated with more aggressive disease as well as shorter overall survival, and was an independent prognostic factor. Additionally, ectopic expression of miR-503 dramatically suppressed cell proliferation and clonogenicity *in vitro* in HCC cells. Furthermore, gain- and loss-of-function studies revealed that miR-503 could block the G1/S transition. Two cell cycle-related molecules, cyclin D3 and E2F3, were further characterized as the direct functional targets of miR-503. Collectively, these findings suggest miR-503 inhibits cell proliferation by cell cycle regulation through Rb-E2F signaling pathways in HCC, providing a potential target for cancer therapy.

## Materials and methods

### Cell lines and tissue specimens

Human embryonic kidney cells (HEK293T),immortalized liver cells (L02) and human HCC cell lines including HepG2, MHCCLM3, MHCC97H, MHCC97L, PLC, HuH7 and Bel-7402 were cultured in Dulbecco’s modified Eagle’s medium with 10% fetal bovine serum at 37°C in a humidified atmosphere containing 5% CO_2_. Paired HCC and adjacent non-tumor liver tissues were collected from patients undergoing liver transplantation (LT) or partial hepatectomy at The First Affiliated Hospital, School of Medicine, Zhejiang University (Hangzhou, P.R. China). Written informed consent was obtained from each patient. A total of 125 patients had a clear histologic diagnosis of HCC with complete clinicopathological data, and all patients were closely followed up for survival analysis. None of the patients received radiotherapy or chemotherapy before surgery. All patients received the same anti-cancer treatment after operation. All sample data were obtained from the clinical and pathologic records and are summarized in Additional file 1: Table S1.

### Oligoribonucleotides

miR-503 mimic, miR-503 inhibitor and the respective control RNA (referred to as NC) were used for the transient gain- and loss-of-function study. The small interfering RNA (siRNA) targeting human cyclin D3 (GenBank Access. No. NM_001136017) and E2F3 (NM_001949) transcripts were designated siCCND3 and siE2F3 respectively. The NC for miR-503 mimic, miR-503 inhibitor and siRNA was non-homologous to any human genome sequence. For the in vivo and in vitro tumorigenicity assay, all nucleotides with 2′-O-methyl modification were used. All the RNA oligoribonucleotides (Additional file 2: Table S2) were purchased from Genepharma (Shanghai, China). All oligoribonucleotides used in this study are shown in Additional file 2: Table S2.

### RNA extraction and quantitative reverse transcriptase polymerase chain reaction (qRT-PCR)

Total RNA from cell lines and clinical samples was isolated using the mirVana miRNA isolation kit (Ambion). Quantitative reverse transcriptase polymerase chain (qRT-PCR) was performed to evaluate the expression level of miR-503 in various cell lines and clinical samples and the expression of cyclin D3 and E2F3 in transfected cells. RNA was reverse transcribed using One Step PrimeScript miRNA cDNA Synthesis Kit (TaKaRa, Japan). The cDNA was then quantified by real-time RT-PCR using SYBR Premix Ex Taq (TaKaRa, Japan). All PCR reactions were performed using the ABI7500 system (Applied Biosystems, CA, USA). RNU6B or glyceraldehyde 3-phosphate dehydrogenase (GAPDH) was used as an internal control, and miR-503 expression values were normalized to RNU6B. All primers used are listed in Additional file 2: Table S2.

### Western blotting

Western blotting was used to detect the expression of target genes at the protein level. Protein was extracted from transfected MHCCLM3 cells using modified RIPA buffer in the presence of proteinase inhibitor cocktail. Equivalent quantities (30–50 μg) of protein were separated in 10% SDS-polyacrylamide gels and transferred to polyvinylidene difluoride membranes. Membranes were blocked with 5% non-fat milk and then incubated overnight at 4°C with the appropriate primary antibody at the dilutions specified by the manufacturer. The membranes were then washed three times in 10-ml TBST and incubated with the corresponding horseradish peroxidase (HRP)-conjugated secondary antibody at 1:2000 dilution for 1 h. Bound secondary antibody was detected using an enhanced chemiluminescence (ECL) system (Pierce Biotechnology Inc., Rockford, IL, USA). Primary antibodies were as follows: anti-E2F3 (Abcam), anti-cyclinD3, anti-Rb, anti-phospho-Ser780-Rb, anti-CDK4, anti-CDK6, anti-cyclin A, anti-cdc2, anti-p15, anti-p16 (Cell Signaling Technology), anti-phospho-Ser811-Rb, and anti-β-actin (Epitomics).

### Cell transfection

The transfections were performed using Lipofectamine 2000 (Invitrogen) according to the manufacturer’s instructions. In brief, MHCCLM3, HepG2, Bel-7402 or HEK293T cells were transfected with DNA, miRNA mimic, miRNA inhibitor, siRNA or respective NC. All RNA transfections were performed at a final concentration of 50 nM. Cells were collected for assay 48 h after transfection. The transfection efficiency of the miR-503 mimic and inhibitor was confirmed by detection of miR-503 expression using qRT-PCR.

### Cell proliferation assay

MHCCLM3, HepG2 and Bel-7402 cells seeded at a density of 4,000 per well into 96-well plates were transfected with miR-503 mimic, miR-503 inhibitor or respective NC. After incubating the cells for the specified time (1, 2, 3 or 4 days), a cell proliferation assay was performed using Cell Counting Kit-8 (CCK-8) (Dojindo) according to the manufacturer’s instructions. The solution absorbance was measured spectrophotometrically at 450 nm with MRX II absorbance reader (Dynex Technologies, Chantilly, VA, USA). The experiments were performed in triplicate.

### Analysis of clonogenicity in vitro and tumorigenicity in nude mice

Aliquots of viable MHCCLM3, HepG2 and Bel-7402 cells (1,000 per well) transfected with miR-503 mimic, miR-503 inhibitor or respective NC were placed in six-well plates 24 h after transfection and maintained in complete medium for 2 weeks. Colony formation was evaluated by staining the cells with 0.1% crystal violet. The rate of colony formation was calculated using the following equation: colony formation rate = (number of colonies/number of seeded cells) × 100*%*. The experiments were performed in triplicate.

Animal study was performed according to institutional ethical guidelines. Male BALB/c-nude mice aged 4 weeks were used for human tumor xenograft model (supplied by the Shanghai Experimental Animal Center, Chinese Academy of Sciences, Shanghai, China). Viable miR-503- or NC-transfected MHCCLM3 cells (5×10^6^) were suspended in 100 μl PBS and then injected subcutaneously into the posterior flank of nude mice, respectively. Tumor growth was examined every week for 5 weeks. Tumor volume (V) was monitored by measuring the length (L) and width (W) with calipers and calculated with the formula (L × W^2^) × 0.5.

### Cell cycle analysis by flow cytometry

Forty-eight hours after transfection, 1×10^5^ MHCCLM3, HepG2 and Bel-7402 cells transfected with miR-503 mimic, miR-503 inhibitor or respective NC were harvested, washed with PBS and fixed in 70% ethanol at 4°C overnight. Staining for DNA content was performed using a DNA Prep Stain (Beckman Coulter, Fullerton, CA, USA). Populations in G0/G1, S and G2/M phases were measured by BD LSRII Flow Cytometry System with FACSDiva software (BD Bioscience, Franklin Lakes, USA). Data were analyzed using the ModFit LT Software. The experiments were performed in triplicate.

### Prediction of miR-503 targets

To predict the target genes and the 3′ UTR binding sites bound by the seed region of miR-503, the TargetScan [8] ( http://www.targetscan.org/), MiRanda [21] ( http://www.microrna.org/microrna/home.do) and PicTar [22] ( http://pictar.mdc-berlin.de/) databases were used. Two cell cycle-related molecules, cyclin D3 and E2F3, were chosen as the direct miR-503 target candidates according to the three databases and the role of miR-503 in cell cycle control.

### Vector construction and luciferase reporter assay

The 3′ UTR of cyclin D3 or E2F3 was amplified by PCR. The amplified product was subcloned and ligated into the pmirGLO Dual-Luciferase miRNA Target Expression Vector (Promega). The recombinant reporter vector was identified by sequencing and termed the wild-type (Wt). To create the miR-503 binding site mutants, the binding region of the seed sequence (5′ GCTGCT 3′) was mutated to the sequence 5′ CGACGA 3′ (mutated nucleotides are in bold and underlined), using the QuikChange Lightning Site-Directed Mutagenesis Kit (Stratagene) according to the manufacturer’s protocol. The recombinant vector was confirmed by sequencing and termed the mutant type (Mut). 293T cells plated in a 24-well plate were co-transfected with 50 nM of either miR-503 mimic or NC and 100 ng of pmirGLO vector comprising Wt or Mut 3′ UTR of cyclin D3 and E2F3 by Lipofectamine 2000. Forty-eight hours after transfection, the relative luciferase activity was measured by Dual-Luciferase Reporter Assay System (Promega) according to the manufacturer’s instructions. All transfection experiments were performed in triplicate.

### Statistical analysis

Data are shown as the means ± SEM of at least three independent experiments. Differences between groups were analyzed using Student’s *t*-test, the χ^2^ test and the log-rank test when two groups were compared. Overall survival rates were calculated actuarially according to the Kaplan–Meier method. Variables with a value of *p* < 0.05 in univariate analysis were used in a subsequent multivariate analysis based on the Cox proportional hazards model. All tests performed were two-sided. A value of *p* < 0.05 was considered to indicate statistical significance.

## Results

### The expression profile of miR-503 in HCC cell lines and tissues and the clinicopathologic significance of miR-503 expression in HCC

**Figure 1:**
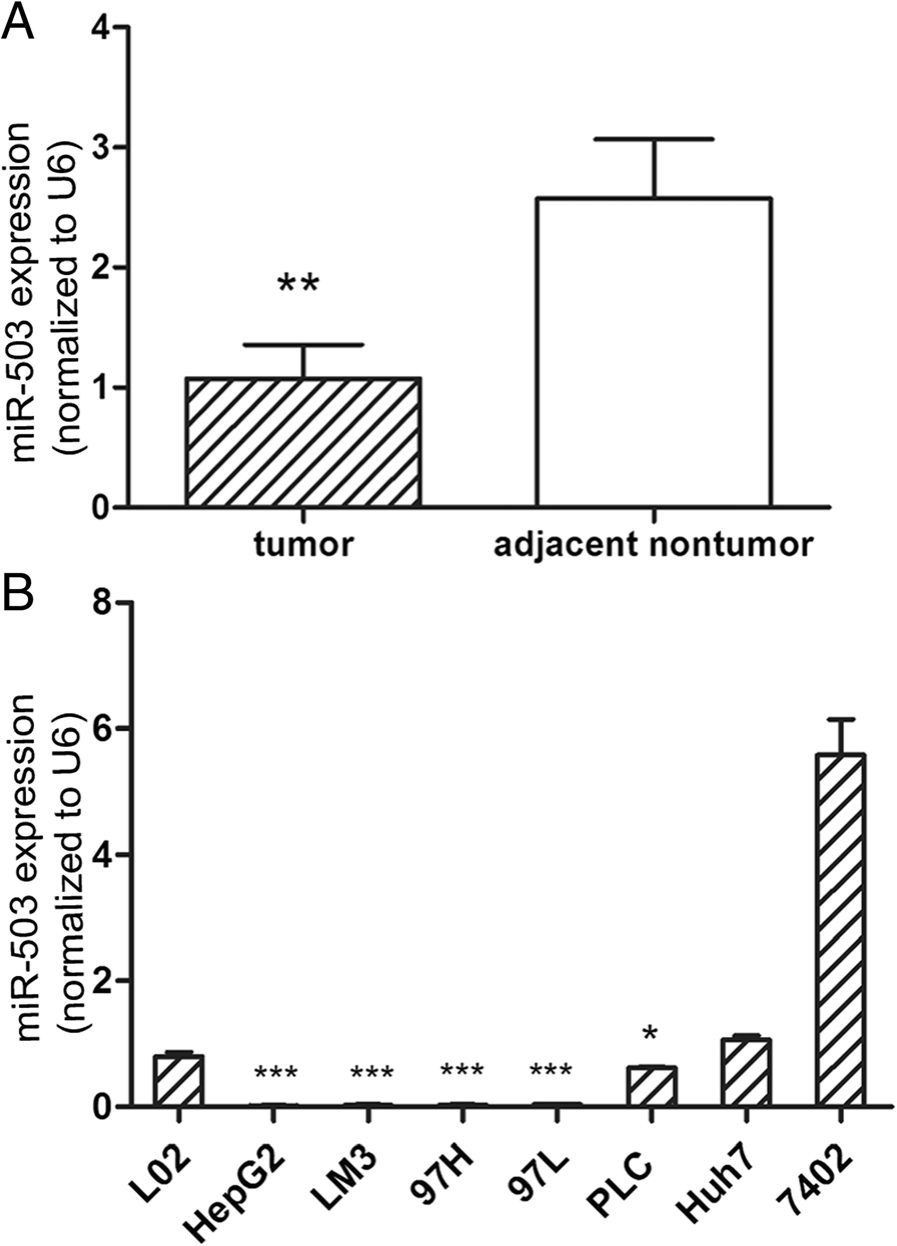
**Expression pattern of miR-503 in HCC cell lines and tissues. (A)** lower expression level of miR-503 was detected in tumors than in adjacent nontumorous tissues in 125 HCC patients. **(B)** relative expression levels of miR-503 in the indicated HCC cell lines (*P<0.05, **P<0.01, ***P<0.001).

To investigate the expression pattern of miR-503 in HCC, we detected the expression of miR-503 in 125 paired HCC and adjacent noncancerous liver tissues by real-time qRT-PCR. Compared with their adjacent non-tumor tissues, significant downregulation of miR-503 was observed in HCC tissues (Figure 1A). Furthermore, the expression of miR-503 was noticeably reduced in five of seven (71.4%) HCC cell lines examined compared with normal liver cell line L02 (Figure 1B). These results suggest that reduced miR-503 expression is a frequent event in human HCC and may be involved in tumorigenesis.

**Figure 2:**
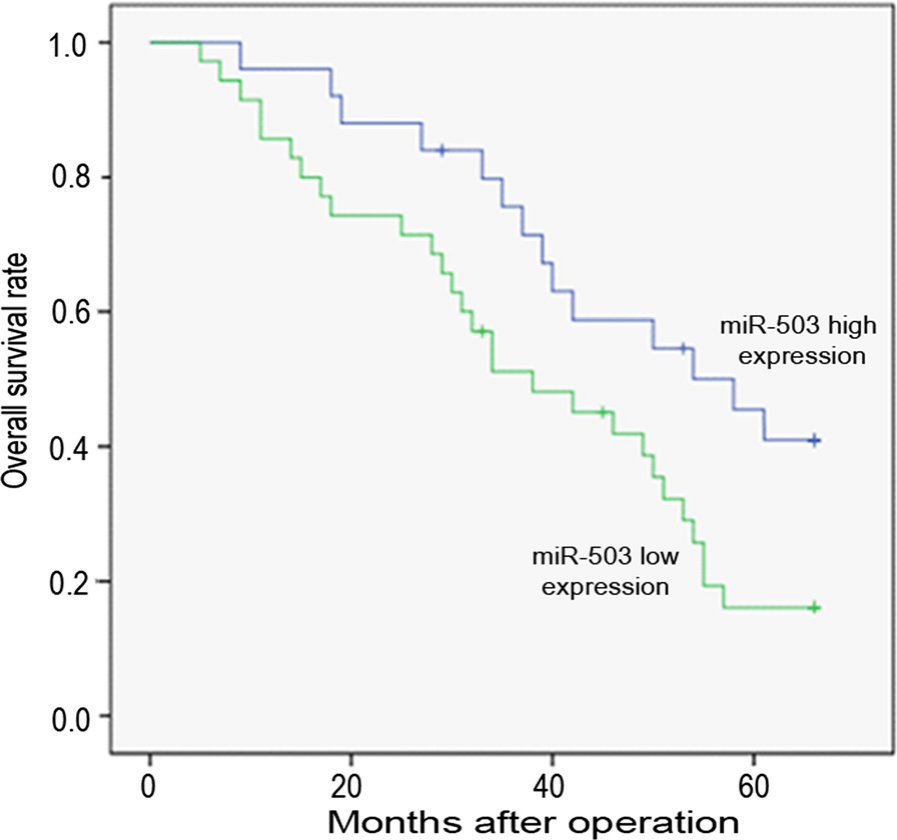
**Kaplan–Meier overall survival curves of HCC patients according to the level of miR-503 expression.** Patients in the low miR-503 expression group had significantly a poorer prognosis than those in the high miR-503 expression group (P<0.01)

We further evaluated the clinicopathologic significance of miR-503 expression in HCC. In this study, patients with expression values less than the average level of miR-503 in HCC tissues (1.75, normalized to RNU6B) were assigned to the low expression group (n=74) whereas patients with values above the average were assigned to the high expression group (n=51). Patients in the low miR-503 expression group had a shorter overall survival than patients in the high miR-503 expression group (*p* = 0.001; Figure 2). Correlation analysis between clinicopathologic factors and miR-503 expression showed that the low miR-503 expression group had later TNM stage (*p* = 0.012), greater AFP levels (*p* = 0.015) and PVTT (*p* = 0.029) than the high miR-503 expression group (Additional file 1: Table S1). In addition, histologic grade in the low miR-503 expression group was poorer than in the high miR-503 expression group (*p* = 0.019). However, no significant differences were observed according to age, sex, HBV, cirrhosis, tumor size and tumor number (Additional file 1: Table S1). The results of univariate and multivariate Cox proportional hazards regression analyses for overall survival are shown in Table 1. Multivariate analysis further confirmed that a high miR-503 expression level was an independent and significant prognostic factor for survival (HR, 2.461; CI, 1.106-4.623; *p* = 0.005; Table 1).

**Table 1:** Univariate and multivariate analysis for overall survival

**Clinical variables**	**HR (95% CI)**	**P-value**
Univariate analysis		
Age	1.021(0.823-1.346)	0.886
Sex	0.953(0.545-1.875)	0.754
HBV	1.067(0.513-2.184)	0.864
Cirrhosis	1.652(0.738-3.678)	0.237
Tumor size	1.542(1.136-2.246)	0.087
Tumor number	2.421(1.546-4.439)	0.246
TNM stage	2.876(1.873-4.786)	**0.002**
Histology grade	1.724(1.120-2.863)	**0.027**
PVTT	3.539(2.238-7.731)	**0.017**
AFP	2.535(1.347-5.042)	**0.027**
miR-503 expression	2.942(1.857-4.974)	**0.001**
Multivariate analysis		
TNM stage	2.054(1.573-4.853)	**0.015**
Histology grade	1.431(0.690-2.968)	0.336
PVTT	0.549(0.242-1.246)	0.152
AFP	1.147(0.575-2.288)	0.697
miR-503 expression	2.461(1.106-4.623)	**0.005**

*HR* Hazard ratios, *CI* confidence interval, *HBV* Hepatitis B virus, *TNM* tumor-node-metastasis, *PVTT* portal vein tumor thrombi, *AFP* Alpha-fetoprotein, Boldface P values indicate statistical significant.

### The effect of miR-503 on cell proliferation, clonogenicity and cell cycle

**Figure 3:**
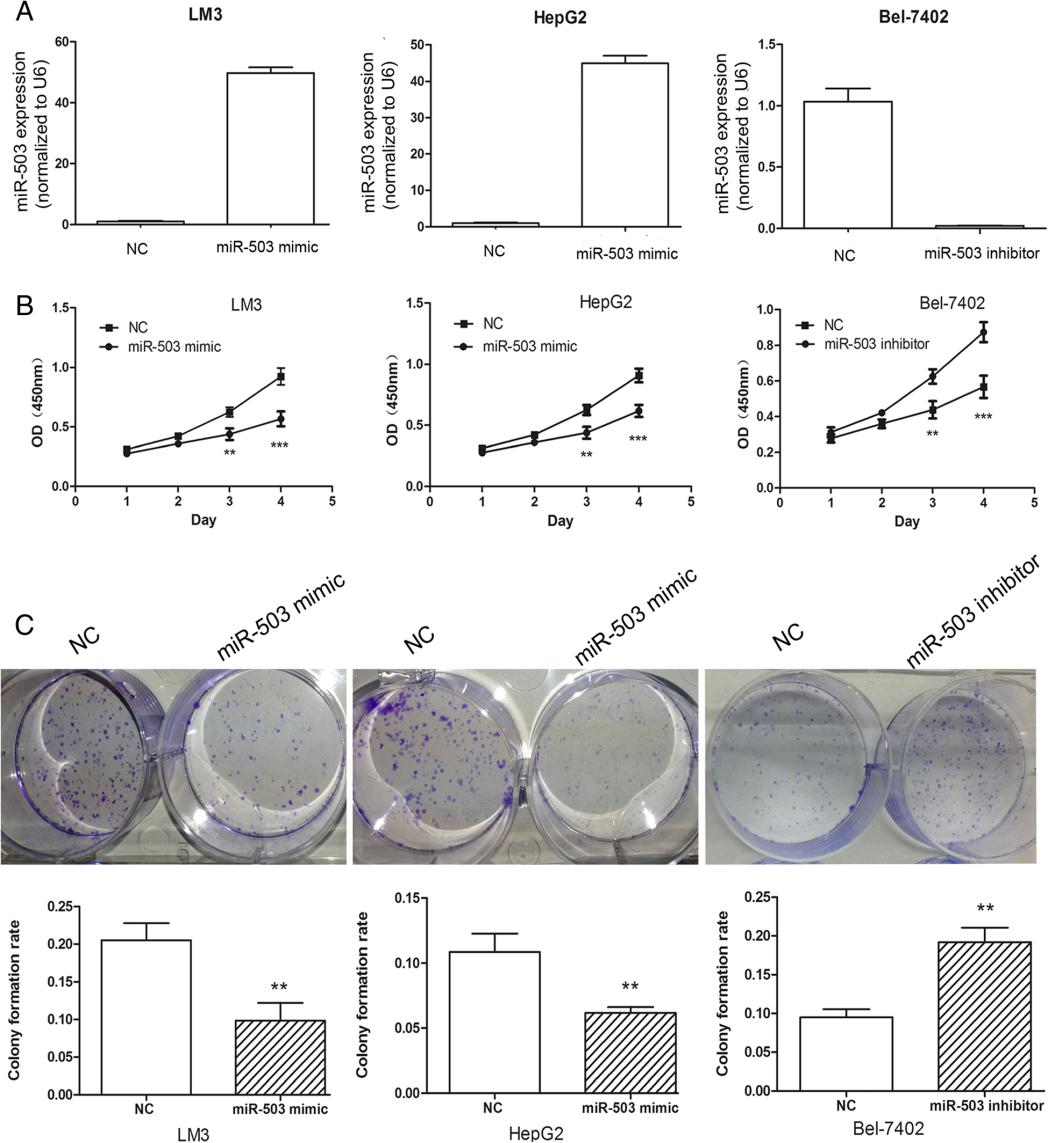
**The effect of miR-503 on cell proliferation, clonogenicity *in vitro*. (A)** The expression of miR-503 in MHCCLM3, HepG2 and Bel-7402 cells with transfection of miR-503 mimic, miR-503 inhibitor or respective NC. **(B)** The effects of miR-503 on the proliferation of HCC cell lines were measured by CCK-8 assay. **(C)** The effect of miR-503 on colony formation of HCC cell lines. **P< 0.01; ***P<0.001.

The frequent downregulation of miR-503 in HCC cell lines and tissues suggests that miR-503 may serve as a tumor suppressor gene (TSG) in HCC. To test the potential tumor suppressor role of miR-503 in HCC, the effect of ectopic miR-503 on cell growth was evaluated in MHCCLM3, HepG2 and Bel-7402 cells transfected with miR-503 mimic, miR-503 inhibitor or respective NC using the CCK-8 cell viability assay. Transfection efficiency of ectopic miR-503 mimic and inhibitor was confirmed by qRT-PCR. The expression of miR-503 was increased 50-folds (MHCCLM3) and 45-folds (HepG2) in cells transfected with 50 nM miR-503 mimic but deceased 40-fold (Bel-7402) in cells transfected with 50 nM miR-503 inhibitor (Figure 3A). At day 1, the OD values of miR-503 mimic, miR-503 inhibitor and respective NC control cells were not significantly different in MHCCLM3, HepG2 or Bel-7402 cells. However, from day 3 onwards, the OD values of MHCCLM3 and HepG2 cells transfected with miR-503 mimic were significantly lower than control cells, but the OD values of Bel-7402 cells transfected with miR-503 inhibitor were significantly higher than control cells (Figure 3B). These data indicate that miR-503 inhibited cell proliferation *in vitro*.

To further investigate the potential role of miR-503 in tumorigenesis, the colony formation assay was performed in MHCCLM3, HepG2 and Bel-7402 cells. Cells were transfected with miR-503 mimic, miR-503 inhibitor or respective NC, and then allowed to grow to a very low density. MHCCLM3 and HepG2 cells transfected with 50 nM miR-503 mimic displayed much fewer and smaller colonies compared with NC duplex transfected cells, but Bel-7402 cells transfected with 50 nM miR-503 inhibitor displayed much more and larger colonies (Figure 3C). These data suggest a growth-inhibitory role of miR-503.

**Figure 4:**
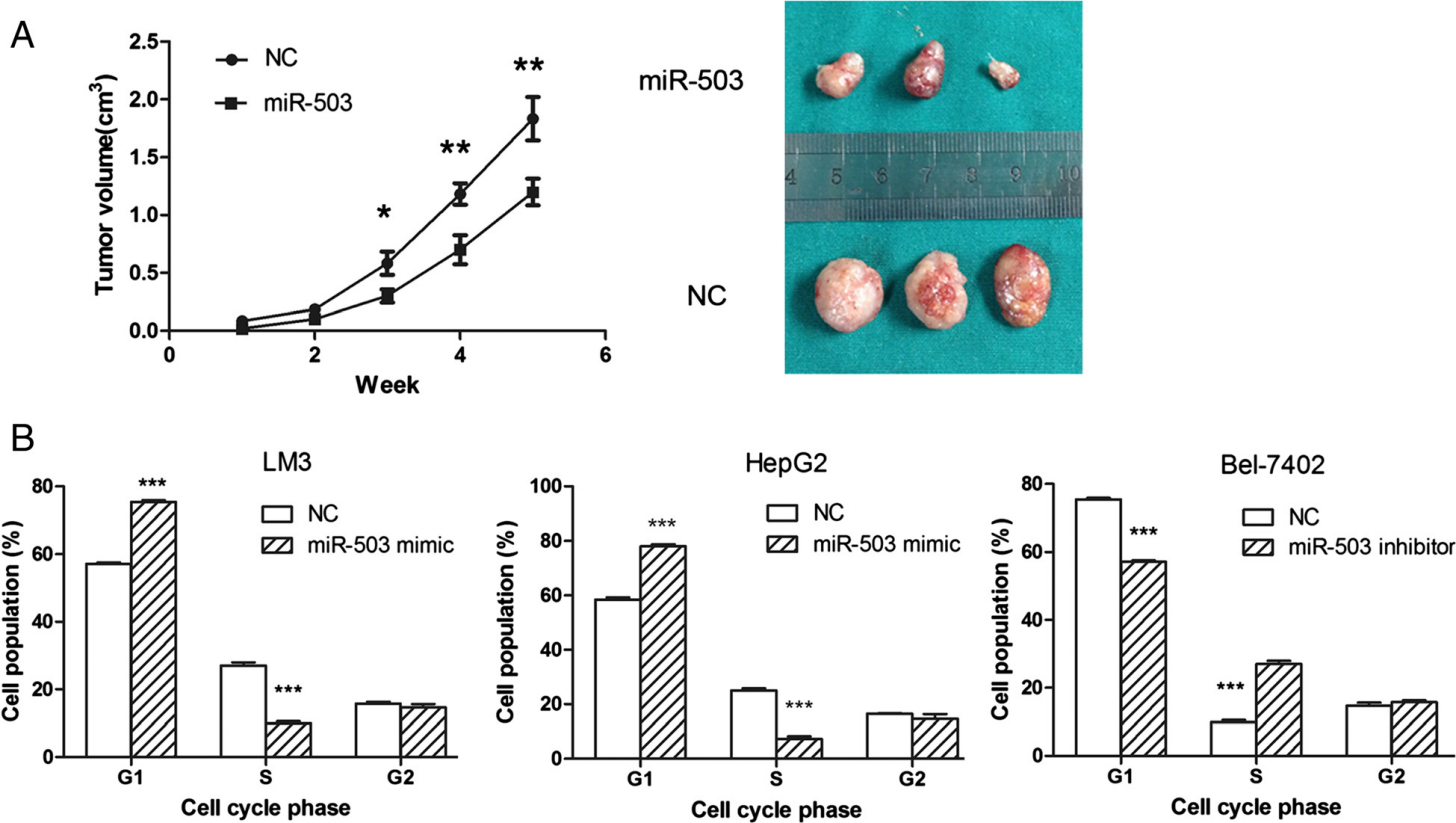
**The effect of miR-503 on clonogenicity *in vivo *and cell cycle. (A)** Tumor formation in nude mice. Representative photographs and the curve of tumor growth are shown. **(B)** The effect of miR-503 on cell cycle of HCC cell lines. *P<0.05 **P< 0.01; ***P<0.001.

To further confirm the above findings, an in vivo mouse model was used. For the duration of the treatment with miR-503 mimic for 5 weeks, tumor volume curves revealed a significant decrease in growth rates at the third, fourth and fifth week after treatment with miR-503 mimic (Figure 4A). Taken together, these results showed that miR-503 inhibits HCC cell growth in vivo and in vitro.

To explore the mechanisms underlying miR-503-suppressed tumor growth, the effect of miR-503 on cell cycle progression was investigated by flow cytometry. The assay showed that the percentages of miR-503 mimic transfected MHCCLM3 and HepG2 cells in the G0–G1 phase were significantly higher than that of NC duplex transfected cells, which paralleled with a significant decrease in the S phase (Figure 4B). Additionally, in miR-503 inhibitor transfected Bel-7402 cells, the percentages of cells in the G0–G1 phase were remarkably lower than that of NC transfected cells, which paralleled with a significant increase in the S phase (Figure 4B). These results indicate miR-503 could inhibit HCC cell proliferation by induction of cell cycle arrest at G1/S phase.

### Cyclin D3 and E2F3 are direct functional targets of miR-503

**Figure 5:**
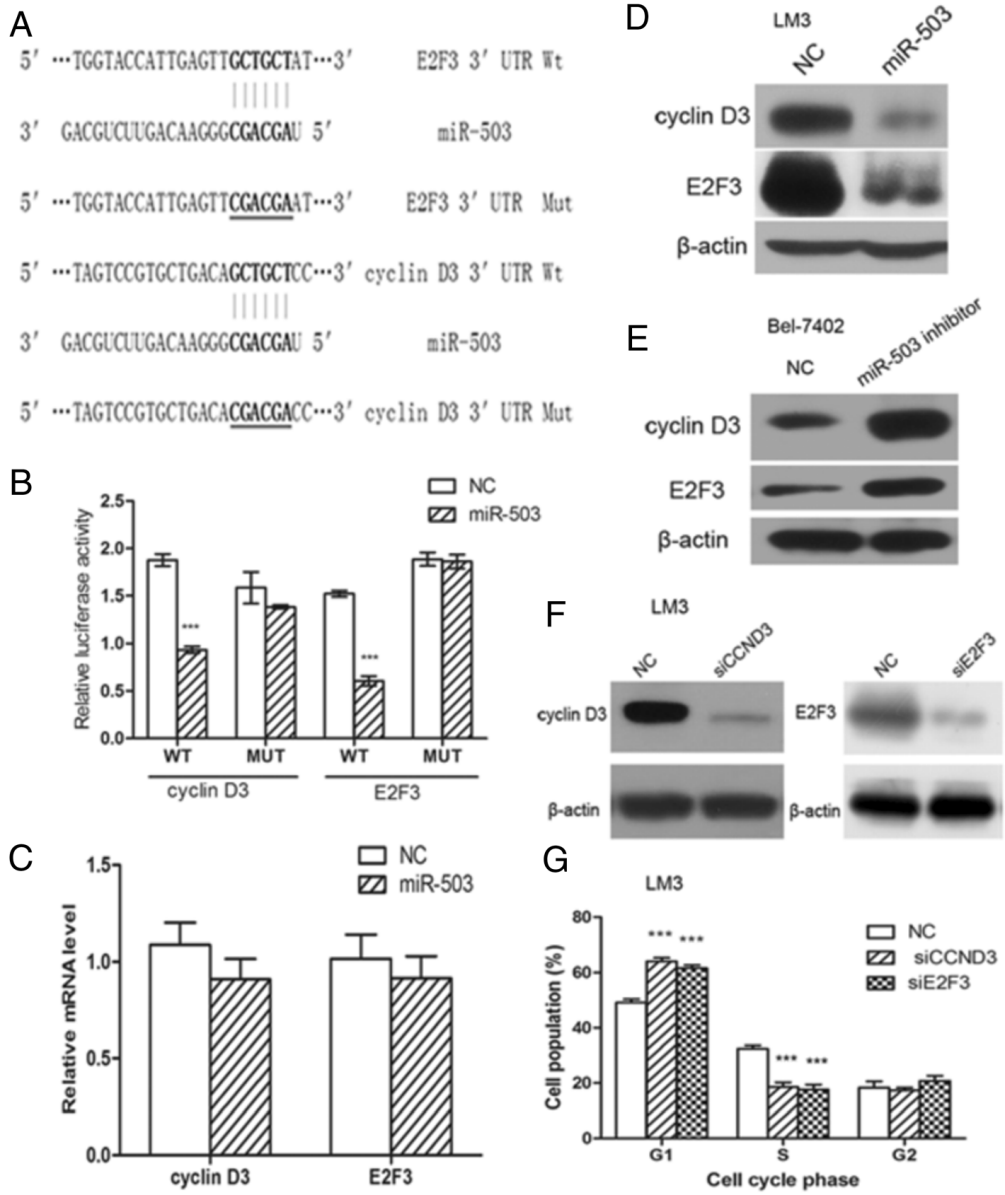
**Cyclin D3 and E2F3 are direct targets of miR-503. (A)** the putative miR-503 binding sequence in the 3′ UTR of Cyclin D3 and E2F3 mRNA. Mutation was generated on the Cyclin D3 and E2F3 3′ UTR sequence in the complementary site for the seed region of miR-503. **(B)** Analysis of luciferase activity. 293 T cells were cotransfected with 50 nM of either miR-503 or NC and 100 ng pmirGLO Vector containing Wt or Mut 3′UTR of Cyclin D3 or E2F3 (indicated as WT or MUT on the X axis). The relative firefly luciferase activity normalized with renilla luciferase was measured 48 h after transfection. **(C)** the effect of miR-503 on the expression of endogenous Cyclin D3 and E2F3 at mRNA levels. **(D** and **E)** Endogenous Cyclin D3 or E2F3 protein levels are down-regulated in LM3 cells transfected with miR-503 mimics and up-regulated in Bel-7402 cells transfected with the miR-503 inhibitor **(F)** the effect of siRNA on the expression of endogenous Cyclin D3 and E2F3 at protein level. **(G)** knockdown of CCND3 or E2F3 induced a significant accumulation of G1-phase cells and blocked G1/S transition. *** P < 0.001.

To further elucidate the molecular mechanism by which miR-503 inhibits the G1/S transition, miRNA target prediction databases, such as TargetScan, miRanda, and PicTar, were employed to predict miR-503 targets. We searched for positive regulators of the G1/S transition among the predicted targets of miR-503. Two cell cycle-related genes, cyclin D3 and E2F3, which are crucial components that initiate the inactivation of the Rb pathway and in turn the G1/S transition, were considered as candidates. A single putative miR-503-binding site was mapped in each 3′ UTR of cyclin-D3 and E2F3 (Figure 5A). To validate whether these genes were direct targets of miR-503, a dual-luciferase reporter system was first employed. PmirGLO Dual-Luciferase miRNA Target Expression Vector containing wild-type or mutant 3′ UTR of the target genes was co-transfected with either NC or miR-503 mimic. Ectopic expression of miR-503 inhibited firefly luciferase activity of the reporter gene with the Wt 3′ UTR but not the Mut reporter gene (Figure 5B), suggesting that miR-503 represses gene expression through its binding sequences at the 3′ UTR of cyclin D3 and E2F3.

In addition, the effect of miR-503 on endogenous expressions of cyclin D3 and E2F3 was assessed by Western blotting. Transfection of miR-503 induced downregulation of cyclin D3 and E2F3 at the protein level in MHCCLM3 cells (Figure 5D) and miR-503 inhibitor significantly up-regulated the expression of cyclin D3 and E2F3 at the protein level in Bel-7402 cells (Figure 5E). However, miR-503 overexpression did not significantly reduce cyclin D3 or E2F3 mRNA levels (Figure 5C), which indicated that miR-503 inhibits the expression of cyclin D3 and E2F3 through translational repression.

Collectively, these data indicate that miR-503 may negatively regulate the expression of cyclin D3 and E2F3 by directly targeting their 3′ UTRs.

### miR-503 was involved in cell cycle regulation through Rb-E2F signaling

To explore the role of cyclin D3 and E2F3 in miR-503-regulated G1/S transition, we investigated whether knockdown of these genes may phenocopy the effect of miR-503 overexpression. MHCCLM3 cells were transfected with siRNA duplex targeting either cyclin D3 or E2F3, which resulted in a significant reduction of the respective genes’ protein levels (Figure 5F). The silencing of either target gene led to G1-phase arrest (Figure 5G), phenocopying the outcome of miR-503 overexpression. Interestingly, compared with miR-503 overexpression, inhibition of cyclin D3 or E2F3 alone caused a less marked G1 arrest, implying that miR-503 may block the G1/S transition by synergistically targeting multiple targets.

**Figure 6:**
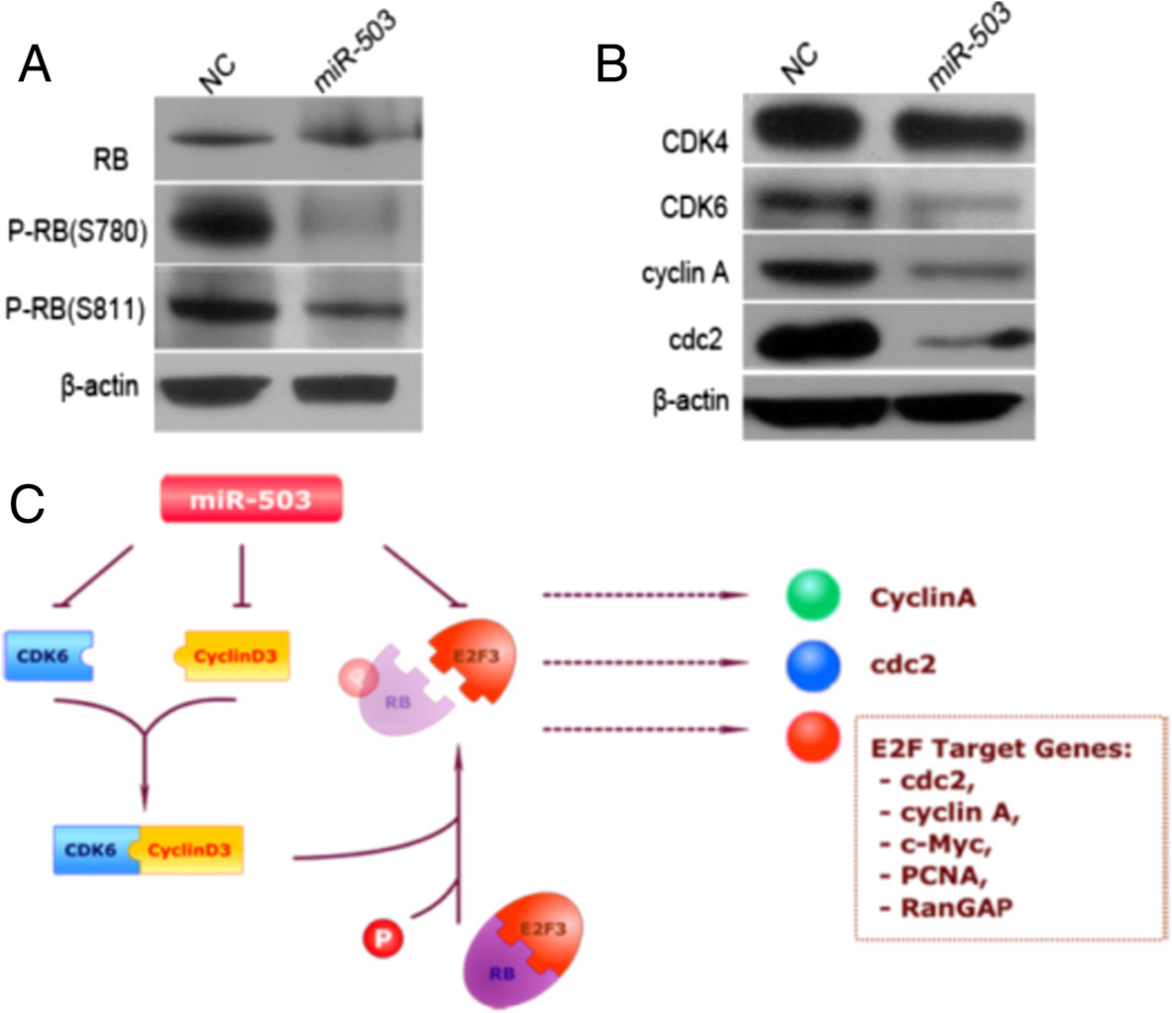
**Rb-E2F signaling is involved in miR-503-regulated G1/S transition. (A)** phosphorylation of Rb protein on Ser780 and Ser811 is inhibited by miR-503. 48 h after transfection with the indicated RNA duplex, cells were subjected to Western blot analysis. Rb, total Rb protein; pRb, phosphorylated Rb. **(B)** effect of miR-503 on the expression of endogenous genes. miR-503 inhibited significantly the expression of CDK6 and the downstream genes of E2F3. Gene expression was examined by Western blot. β-actin, internal control. **(C)** signaling pathways that regulate the cell cycle control in HCC involving miR-503.

Cyclin D3 and CDK6 are crucial molecules that initiate the phosphorylation of retinoblastoma (Rb), which results in the release of E2F and subsequently the transactivation of genes required for S-phase entry. Therefore, we investigated whether miR-503 could attenuate these events. Ectopic expression of miR-503 caused a reduction of phosphorylated Rb protein, although little effect on the total Rb protein was found (Figure 6A). Moreover, the effect of miR-503 on endogenous genes such as CDK4, CDK6, p15 and p16, was further investigated. Overexpression of miR-503 induced significant downregulation of CDK6; however, little effect on endogenous expression of p15, p16 or CDK4 was observed (Figure 6B). Furthermore, the protein levels of E2F downstream targets, including cdc2 and cyclin A genes, were significantly lower in miR-503-transfected MHCCLM3 cells than in NC transfectants (Figure 6B).

These data suggest that miR-503 may inhibit the G1/S transition by directly suppressing both cyclin D3 and E2F3 through Rb-E2F signaling pathways (Figure 6C).

## Discussion

Accumulating evidence has revealed that deregulation of cell cycle control is an essential step in carcinogenesis [23,24]. Recent studies have indicated that miRNAs are involved in tumorigenesis through the regulation of the cell cycle. Wang *et al*. reported miR-138 induces cell cycle arrest by targeting cyclin D3 in HCC [25]. Therefore, deregulation of cell cycle-related miRNAs may facilitate tumorigenesis. Deregulation of miR-503 has been identified in various disease types, including cancers [19,26-31]. Caporali *et al*. reported that overexpression of miR-503 inhibited endothelial cell proliferation and migration by targeting CCNE1 and cdc25A [26]. Ectopic miR-503 expression induced G1 arrest by directly targeting cell-cycle regulators in acute myeloid leukemia [27]. Reportedly, miR-503 suppressed the endogenous CCND1 both at protein and mRNA levels by binding to the 3′ UTR of the CCND1 gene and inhibited cell growth by reducing S-phase cell populations in human head and neck carcinomas [28]. miR-503 induced G1 arrest by downregulating cdc25A in heterologous cancer cells [30]. However, the molecular mechanisms by which miR-503 modulates cell cycle control in HCC are still largely unknown.

Our findings showed that miR-503 downregulation was a frequent event in human HCC tissues. However, miR-503 was highly expressed in retinoblastoma [31]; this discrepancy may be due to use of different cell lines. Gain- and loss-of-function studies indicated overexpression of miR-503 could suppress cell proliferation and colony formation and induce cell cycle arrest in HCC. We propose that reduced expression of miR-503 may disrupt cell cycle control, subsequently promote cell proliferation, and consequently facilitate the development and progression of HCC. These results suggest the important role of miR-503 in HCC tumorigenesis.

A family of the CDKs and their activating partners (cyclins) are involved in regulation of the cell cycle. The G1/S phase transition is regulated primarily by D-type cyclins (D1, D2 or D3) in complex with CDK4/CDK6. These complexes cooperate in phosphorylating proteins of the Rb family and leading to release of E2F transcription factors, thus activating E2F-mediated transcription and driving cells from G1 into S phase. The Rb pathway is known to act as a key checkpoint in cell cycle progression. Our results showing that miR-503 targets molecules both upstream (cyclin D3) and downstream (E2F3) of Rb, an archetypal tumor suppressor, provide new insight into tumorigenesis. Further investigations revealed that miR-503 significantly inhibits the expression of CDK6 at the protein level. Whether miR-503 directly suppresses CDK6 expression requires further study. Collectively, our data suggest miR-503 is involved in the cell cycle regulation through Rb-E2F signaling in HCC.

The underlying mechanism responsible for the decreased expression of miR-503 in HCC remains unclear. More than half of the annotated human miRNA genes are located in fragile sites in genomic regions that are frequently amplified, deleted, or rearranged in human cancers [32], providing plausible mechanisms of reduced miR-503 expression. In addition, promoter hypermethylation or transcriptional regulation might account at least in part for the reduced miR-503 expression in HCC. Further investigation is required to verify this hypothesis.

In summary, our data suggest that miR-503 functions as a powerful tumor suppressor and could be an independent prognostic marker in HCC patients. Moreover, the frequently downregulated miR-503 can inhibit the G1/S phase transition by directly targeting cyclin D3 and E2F3 through Rb-E2F signaling pathways. Therefore, miR-503 may serve as a useful therapeutic target for an miRNA-based HCC therapy.

## Competing interests

All authors declared that they have no competing interest.

## Authors’ contributions

LZ and SSZ contributed to the conception and design of the study. FQX and WZ performed the experimental work. FC and HYX interpreted the data and helped to draft the manuscript. LMC participated in the design of the study. HJG and JC performed the statistical analysis. CYX, XBY, SMD and KJC participated in the experiments. All the authors contributed to drafting and reviewing the manuscript, and all authors read and approved the final manuscript.

## Supplementary Material

Additional file 1: Table S1 Correlation between the expression level of miR-503 and clinicopathological parameters. Click here for file

Additional file 2: Table S2 List of the oligonucleotides used in this study. Click here for file
