# Supplementary material for: MicroRNA-503 inhibits the G1/S transition by downregulating cyclin D3 and E2F3 in hepatocellular carcinoma
Source: J Transl Med. 2013 Aug 22;11:195. doi: 10.1186/1479-5876-11-195 (PMC3765277; doi:10.1186/1479-5876-11-195)
Supplement: Additional file 1: Table S1 — Correlation between the expression level of miR-503 and clinicopathological parameters. [file 1479-5876-11-195-S1.doc]

| Additional Table s1. Correlation between the expression level of miR-503 and clinicopathological parameters  Low expression High expression  group(n=74) group(n=51)  Factors n % n % ^a^P  Age(years)  ≤50 34 45.9 23 45.1 0.925  ＞50 40 54.1 28 54.9  Sex  Male 54 73.0 35 68.6 0.598  Female 20 27.0 16 31.4  HBV  Positive 59 79.7 39 76.5 0.663  Negative 15 20.3 12 23.5  Cirrhosis  Positive 57 77.0 37 72.5 0.569  Negative 17 23.0 14 27.5  Tumor size  ≤5cm 48 64.9 32 62.7 0.871  ＞5cm 26 35.1 19 37.3  Tumor number  Single 45 60.8 30 58.8 0.824  Multiple 29 39.2 21 41.2  Histologic grade  Well and moderately 21 28.4 25 49.0 0.019  Poor and others 53 71.6 26 51.0  TNM stage  Ⅰ 25 33.8 32 62.7 0.012  Ⅱand Ⅲ 49 66.2 19 37.3  AFP( ng/ml)  ≤400 30 40.5 32 62.7 0.015  ＞400 44 59.5 19 37.3  PVTT  Positive 28 37.8 10 19.6 0.029  Negative 46 62.2 41 80.4  PVTT, portal vein tumor thrombi; AFP, α-fetoprotein; ^a^Chi-square test (Pearson Chi-square) |
| --- |
