# Supplementary material for: MicroRNA-503 inhibits the G1/S transition by downregulating cyclin D3 and E2F3 in hepatocellular carcinoma
Source: J Transl Med. 2013 Aug 22;11:195. doi: 10.1186/1479-5876-11-195 (PMC3765277; doi:10.1186/1479-5876-11-195)
Supplement: Additional file 2: Table S2 — List of the oligonucleotides used in this study. [file 1479-5876-11-195-S2.doc]

| Additional Table s2. List of the oligonucleotides used in this study.  Name^a^  Sequence( 5＇ 3＇)^b^  **miRNA and siRNA Duplexes**  miR-503 mimic(sense) UAGCAGCGGGAACAGUUCUGCAG  miR-503 inhibitor(sense) CUGCAGAACUGUUCCCGCUGCUA  NC(sense) ACUACUGAGUGACAGUAGA[dT][dT]  SiCCND3(sense) GGGACAGAAUUGGAUACAUTT  SiE2F3(sense) GCACUACGAAGUCCAGAUATT  **Primers for RT-PCR**  CCND3 F GATGGGACAGAATTGGATAC  CCND3 R GAGCATTTTGGCAGTTGA  E2F3 F AGACTTGAAGTGCCTGACTC  E2F3 R GAAGCGGGTTTAGGGATA  GAPDH F AAGGTGAAGGTCGGAGTCA  GAPDH R GGAAGATGGTGATGGGATTT  **Primers for 3'UTR Cloning**  CCND3-utr F CG**GAGCTC**TCTAGGGTTATTGCATTTGG  CCND3-utr R CGC**GTCGAC**CAGAGGGACAATGGGAGA  CCND3-mut F aggcccctatgtagtccgtgctgaca**cgacga**cctagaggg  agggg  CCND3-mut R cccctccctctaggtcgtcgtgtcagcacggactacatagg  ggcct  E2F3-utr F CG **GAGCTC** CTGGGTTTAACTGGTGTA  E2F3-utr R CGC**GTCGAC**ATGAAAACAAGAGCCACA  E2F3-mut F atgcaggtttcctggtaccattgagtt**cgacga**attaaagct  cacacacgaaatggctaa  E2F3-mut R ttagccatttcgtgtgtgagctttaattcgtcgaactcaatgg  taccaggaaacctgcat  ^a^ F, forward primer; R, reverse primer.  ^b^ Restriction sites are in bold; Mutated sites are bold and underlined. |
| --- |
